# Evaluation of factors predicting the benefit from systemic oncological treatment for severely ill hospitalized patients: a retrospective study

**DOI:** 10.1186/s12904-023-01256-8

**Published:** 2023-09-06

**Authors:** Milena Brachmans Mascarenhas Neves, Yuri Costa Sarno Neves, Juliana Vieira Biason Bomonetto, Priscila Prais Carneiro Matos, Auro Del Giglio, Daniel de Iracema Gomes Cubero

**Affiliations:** 1 Centro Universitário Faculdade de Medicina do ABC (FMABC), Santo André, SP Brazil; 2 Hospital Alemão Oswaldo Cruz, 212. Vila Mariana, 0412601 São Paulo, SP Brazil; 3 Instituto de Radiologia (InRad), Faculdade de Medicina, Hospital das Clinicas HCFMUSP, Universidade de Sao Paulo, Sao Paulo, SP Brazil

**Keywords:** Patients with cancer, Chemotherapy, Systemic oncological treatment, Palliative care, Hospital medicine, ECOG, End of life

## Abstract

**Background:**

Patients with cancer in the disease’s end-stage with poor performance represent a challenging clinical scenario, as they have high chance of a fatal outcome due to clinical conditions, oncological emergencies, and/or metastatic disease. This study examines the factors predicting the potential benefit of “urgent” chemotherapy during hospitalization in this setting, thus addressing a research gap.

**Methods:**

This retrospective observational study was conducted in the largest cancer center in the outskirts of São Paulo. It identified factors predicting the benefit from antineoplastic treatment in severe in-hospital patients admitted during 2019–2020, considering post-chemotherapy survival time as the main dependent variable. Data were retrieved from medical records. All patients aged ≥ 18 years, with an ECOG-PS score ≥ 2, and undergoing non-elective systemic cancer treatment were included.

**Results:**

This study evaluated 204 records, of which 89 were included in the final analysis. A statistically significant association with the worse outcome (death within 30 days of chemotherapy) was found with higher ECOG performance status; chemotherapy dose reduction; lower values of serum albumin, hemoglobin, and creatinine clearance; and higher values of leukocytes, neutrophils, direct bilirubin, urea, and C-reactive protein. In the multivariate analysis, only albumin remained statistically associated with the outcome (hazard ratio = 0.35; confidence interval: 0.14, 0.90; p = 0.034).

**Conclusions:**

Serum albumin and other clinical and laboratory variables might be associated with early post-treatment deaths in patients with cancer. The study data might help guide the decision to administer systemic treatment in this scenario and manage critically ill patients. This study adds to our knowledge of the factors predicting the objective benefits from “heroic” or “urgent” chemotherapy for hospitalized and severely ill patients with cancer.

## Background

More than 625,000 new cases of cancer are diagnosed in Brazil annually [1]. Many of them, due to early diagnosis, are considered cured or undergo remission for long periods with currently available treatments; meanwhile, others are diagnosed at advanced or metastatic stages, making effective and curative treatment difficult or even unfeasible.

Owing to the natural history of cancer and the healthcare system’s access inequalities, we often encounter patients with advanced disease or poor clinical performance. Hence, we may find patients with end-stage cancer who have undergone several lines of treatment, with years of disease evolution; we may also come across patients with low performance status but with a recent diagnosis and no opportunity yet to be evaluated by a specialist and receive adequate cancer treatment [2–5]. It is known that patients with cancer who are hospitalized tend to be more symptomatic and in a more advanced stage of the disease than patients undergoing outpatient treatment and follow-up [6]. One of the biggest challenges in oncology practice is knowing which patients can benefit from urgent oncological treatment when hospitalized due to a severe clinical condition with a high risk of death, because of the cancer itself or its related complications.

Previous studies have demonstrated that cytotoxic chemotherapy increases survival and quality of life compared with exclusive palliative care in patients with advanced cancer [7, 8]. However, some authors consider that performing chemotherapy or invasive procedures that do not add to the quality or duration of life, especially in the last weeks, is a criterion of low-quality care for patients with cancer; therefore, it should be avoided whenever possible [8]. Nevertheless, it is important to carry out a careful, objective assessment that can help us predict who can benefit from urgent oncological treatment when hospitalized in a severe condition and for whom such intervention would result in only prolonging suffering for themselves and their families. Unfortunately, to the best of our knowledge, there are no robust predictive prognostic biomarkers [7] nor prognostic scales that can numerically and objectively determine whether a patient is a good candidate for chemotherapy in this setting. Thus, this study aimed to identify factors predicting the probability of antineoplastic treatment benefitting hospitalized patients with advanced-stage disease and poor clinical performance, and to evaluate the clinical outcomes of these patients undergoing chemotherapy, including oncological emergencies such as visceral liver or other organ crisis, intestinal subocclusion, superior vena cava syndrome, and medullary compression syndrome.

## Methods

### Study design and the inclusion and exclusion criteria

This retrospective observational study was conducted from January 1, 2019, to December 31, 2020, at the Hospital de Clínicas de São Bernardo do Campo, São Paulo, Brazil. The medical records of all hospitalized patients with a diagnosis of advanced solid cancer who underwent cancer treatment were evaluated. All patients aged 18 years with an Eastern Cooperative Oncology Group Performance Status Scale (ECOG-PS) score of 2–4 and undergoing systemic cancer treatment (chemotherapy or hormone therapy) during hospitalization were included in the study. Indications for urgent systemic treatment took into account criteria such as oncological urgencies, patient symptomatology, uncontrolled disease (risk of rapid progression) and number of previous lines of chemotherapy; final decision was subjective (at discretion of the attending oncologist). Patients hospitalized for elective chemotherapy were excluded.

The primary outcome was death within 30 days post-systemic therapy. The last available time point was considered as the last outpatient visit or death.

### Statistical analysis

For descriptive purposes, categorical variables were presented as relative and absolute frequencies. Normally distributed continuous data were presented as the means and standard deviations or as medians and quartiles. The normality assumption was assessed using skewness and kurtosis values, as well as graphical methods.

For the binary early outcomes of death or survival within 30 days, categorical variables were compared using the chi-square test, and continuous variables were compared using an independent samples t-test or Mann-Whitney U test, as appropriate. Age, body-mass index (BMI), and ECOG score were included a priori in the multivariable models. Additional variables with a univariate p < 0.10 were included in the multivariable Cox regression model, and the results were presented as hazard ratios (HR) and 95% confidence intervals (CI).

Multiple imputations were performed for missing data before the multivariate analysis. The following variables included in the multivariable models had missing cases imputed: direct bilirubin (25 missing cases), albumin (51 missing cases), and C-reactive protein (1 missing case).

All tests were two-tailed, and statistical significance was set at p < 0.05. All analyses were conducted using the STATA software (StataCorp, 2021; Stata Statistical Software: Release 17, College Station, TX, StataCorp LLC).

## Results

**Fig. 1:**
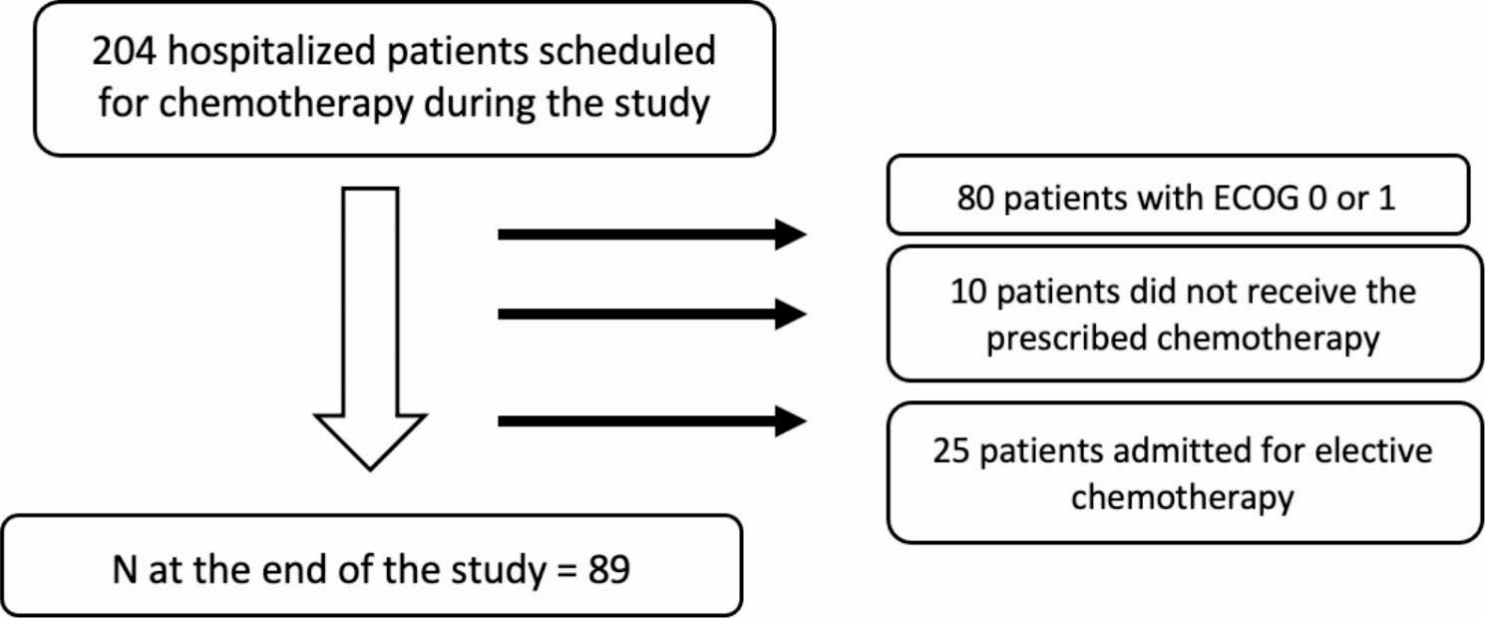
Study flowchart. ECOG = Eastern Cooperative Oncology Group Performance Status, N = number of patients

The medical records of 204 patients who received in-hospital systemic cancer treatment during the study period were evaluated. Of these, 80 patients were excluded because they had ECOG score of 0 or 1, 10 were excluded because they did not receive the prescribed chemotherapy, and 25 were excluded because they were admitted for elective chemotherapy. Of the initial 204 participants, 89 were included in the analysis. Figure 1 presents a flowchart of patient enrollment.

Moreover, 48 (53.9%) patients were women with a mean age of 59.0 years (standard deviation: 12.1). Most patients were white (56.1%) and had an elementary school education (45.3%). Table 1 presents all descriptive demographic and clinical data of the study population.

**Table 1:** Demographic and clinical characteristics (descriptive statistics)

Variable	Overall, N = 89 (%)
**Age (years)** (mean ± SD) Range	59.0 ± 12.1 25–81
**Female**	48 (53.9%)
**BMI** (kg/m^2^) (mean ± SD)	23.5 ± 6.7
**Race**	
- White	50 (56.1%)
- Black	6 (6.7%)
- Brown	32 (35.9%)
**Schooling**	
- Complete high school	19 (35.8%)
- Incomplete high school	2 (3.8%)
- Elementary school	24 (45.3%)
- Graduation	3 (5.7%)
**Comorbidity**	73 (82%)
- Smoking	45 (50.6%)
- Alcoholism	19 (21.4%)
- Diabetes mellitus	22 (24.7%)
- Hypertension	42 (47.2%)
- Heart	4 (4.5%)
- Pulmonary	9 (10.1%)
- Renal	8 (8.9%)
- Liver	3 (3.4%)
- Autoimmune	10 (11.2%)
- Degenerative orthopedic	4 (4.5%)
**ECOG**	
- 2	44 (49.4%)
- 3	42 (47.2%)
- 4	3 (3.4%)
**Cancer**	
- Lung	23 (25.8%)
- Breast	15 (16.9%)
- Colon	9 (10.1%)
- Ovary	8 (9.0%)
- Stomach	5 (5.6%)
- Prostate	4 (4.5%)
**Stage T**	
- 1	2 (2.3%)
- 2	9 (10.1%)
- 3	11 (12.4%)
- 4	38 (42.7%)
- Tx	29 (32.6%)
**Stage N**	
- 0	4 (4.5%)
- 1	19 (21.4%)
- 2	10 (11.2%)
- 3	8 (9.0%)
- Nx	48 (53.9%)
**Distance metastasis (M1)**	86 (96.6%)
**Number of metastasis Sites**	
- 1	29 (32.6%)
- 2	31 (34.8%)
- 3	17 (19.1%)
- 4 or more	9 (10.1%)
**Metastasis sites**	
- Lung	40 (44.9%)
- Liver	36 (40.4%)
- Lymph node	28 (31.5%)
- Bone	23 (25.8%)
- Peritoneum	20 (22.47%)
- Pleura	15 (16.85%)
- Central nervous system	9 (10.11%)
- Skin	3 (3.37%)
- Adrenal gland	3 (3.37%)
- Bone marrow	3 (3.37%)
**Clinal stage**	
- 2	1 (1.1%)
- 3	2 (2.2%)
- 4	86 (96.6%)
**Previous chemotherapy lines**	
- 0	70 (78.7%)
- 1	11 (12.4%)
- 2 or more	8 (8.9%)
**Oncological emergency**	38 (42.7%)
- Hepatic visceral crisis	9 (23.7%)
- Intestinal subocclusion	8 (21.1%)
- Superior vena cava syndrome	7 (18.4%)
- Spinal cord compression syndrome	7 (18.4%)
- Pulmonary lymphangitis	6 (15.8%)
- Hypercalcemia	1 (2.6%)
**Purpose of systemic therapy**	
- Neoadjuvant or adjuvant	2 (2.2%)
- Palliative	87 (97.8%)
**Type of systemic oncological treatment**	
- Chemotherapy	83 (93.3%)
- Hormone therapy	6 (6.7%)
**Reduced chemotherapy dose**	18 (20.2%)
**Other inpatient treatments**	
- Radiotherapy	9 (10.2%)
- Surgery	4 (4.6%)
**Assessment by palliative care team**	30 (33.7%)
**Death** (until last assessment)	75 (84.3%)
**Death within 30 days after chemotherapy**	52 (58.4%)
**Post-chemotherapy survival** (days) (mean ± SD)	74.6 ± 148.1
- Spread	1–863
**Immediate cause of death**	
Cancer	53 (73.6%)
Septic shock	6 (8.3%)
Febrile neutropenia	5 (6.9%)
Tumor lysis syndrome	4 (5.6%)
Respiratory failure	1 (1.4%)
Hypovolemic shock	3 (4.2%)
**ICU admission**	18 (20.2%)
**Performing orotracheal intubation / mechanical ventilation**	16 (18.0%)
**Blood transfusion**	13 (14.6%)
**Central venous catheter insertion**	29 (32.6%)

Notes: Data are shown as n (%) unless otherwise specified. SD = standard deviation, BMI = body-mass index, ECOG = Eastern Cooperative Oncology Group Performance Status, T = tumor size, N = number of lymph nodes, M = presence or absence of distant metastases, ICU = intensive care unit

Regarding clinical performance, 44 patients (49.4%) were classified as ECOG 2, 42 (47.2%) as ECOG 3, and three (3.37%) as ECOG 4. Among the 89 included patients, 17 different types of primary solid neoplasia were identified, of which the five most prevalent types were lung cancer (25.8%), breast cancer (16.8%), colon cancer (10.1%), ovarian cancer (9%), and stomach cancer (5.6%). As for tumor (T), nodal (N), and metastatic (M) staging [8] at the time of admission, 38 (42.7%) patients were classified as T4, 48 (53.9%) patients did not have an N descriptor assignment in the medical record (Nx), and the majority (96.6%) were metastatic. Thirty-eight (42.7%) patients had some type of oncological emergency that led to hospitalization or that occurred during hospitalization.

Regarding the type of oncological treatment received during hospitalization, 83 (93.3%) patients underwent systemic chemotherapy, and 6 (6.7%) patients received hormone therapy. A total of 78.7% of the patients had never received previous lines of chemotherapy. Moreover, 12.4% had received one previous line of chemotherapy, and 8.9% had received two or more lines of chemotherapy. A systemic treatment scheme was prescribed with a reduced dose for 18 (20.2%) patients; clinical judgments and decisions were made by the attending physician at the time of prescription, mainly considering the patients’ performance status. During hospitalization, 13 (14.6%) patients required additional oncological treatment: radiotherapy (n = 9) and surgery (n = 4). Further, 30 (33.7%) were evaluated by a palliative care team during hospitalization, and 58 (65.2%) received chemotherapy during hospitalization as the last oncological treatment before death or until their last medical outpatient evaluation (after hospital discharge).

Seventy-five (84.3%) patients died during the study period, of which fifty-two (58.4%) died within 30 days of systemic therapy and 36 (40.4%) died by two weeks; main causes were neoplasia (73.6%), septic shock (8.33%), and febrile neutropenia (6.9%). Post-chemotherapy survival time ranged from 1 to 863 days, with a mean of 74.6 days (standard deviation: 148) (Table 1). Table 2 describes the bivariate analyses of the categorical variables (demographic, clinical, and laboratory characteristics).

**Table 2:** Association between demographic-clinical, and laboratory characteristics and death within 30 days post-systemic oncological therapy (bivariate analysis)

Characteristic	Overall, N = 89^1^	Survival of at least 30 days after therapy, N = 37^1^	Death within 30 days after therapy, N = 52^1^	p-value^2^
**Age** (years)	59.28 (12.23)	58.44 (11.55)	59.89 (12.76)	0.578
**Male**	41 / 89 (46%)	14 / 37 (38%)	27 / 52 (52%)	0.189
**Race**				0.481
White	50 / 89 (56%)	18 / 37 (49%)	32 / 52 (62%)	
Black	6 / 89 (7%)	3 / 37 (8%)	3 / 52 (6%)	
Brown	32 / 89 (36%)	16 / 37 (43%)	16 / 52 (31%)	
Asian	1 / 89 (1%)	0 / 37 (0%)	1 / 52 (2%)	
**Schooling**				0.541
Complete high school	19 / 53 (36%)	7 / 23 (30%)	12 / 30 (40%)	
Incomplete high school	2 / 53 (4%)	0 / 23 (0%)	2 / 30 (7%)	
Elementary school I	12 / 53 (23%)	6 / 23 (26%)	6 / 30 (20%)	
Elementary school II	12 / 53 (23%)	7 / 23 (30%)	5 / 30 (17%)	
Graduation	2 / 53 (4%)	1 / 23 (4%)	1 / 30 (3%)	
Incomplete higher	1 / 53 (2%)	1 / 23 (4%)	0 / 30 (0%)	
Illiterate	5 / 53 (9%)	1 / 23 (4%)	4 / 30 (13%)	
(Missing data)	36	14	22	
**Comorbidity**	73 / 89 (82%)	30 / 37 (81%)	43 / 52 (83%)	0.845
Heart	4 / 89 (4%)	2 / 37 (5%)	2 / 52 (4%)	> 0.999
Pulmonary	9 / 89 (10%)	5 / 37 (14%)	4 / 52 (8%)	0.481
Renal	8 / 89 (9%)	3 / 37 (8%)	5 / 52 (10%)	> 0.999
Liver	3 / 89 (3%)	0 / 37 (0%)	3 / 52 (6%)	0.263
Autoimmune	10 / 89 (11%)	5 / 37 (14%)	5 / 52 (10%)	0.736
Degenerative orthopedic	4 / 89 (4%)	0 / 37 (0%)	4 / 52 (8%)	0.138
Vascular				> 0.999
Deep vein thrombosis	3 / 7 (43%)	2 / 5 (40%)	1 / 2 (50%)	
Aortic aneurysm	2 / 7 (29%)	1 / 5 (20%)	1 / 2 (50%)	
Stroke	2 / 7 (29%)	2 / 5 (40%)	0 / 2 (0%)	
(Missing data)	82	32	50	
**ECOG**				**< 0.001**
2	44 / 89 (49%)	27 / 37 (73%)	17 / 52 (33%)	
3	42 / 89 (47%)	10 / 37 (27%)	32 / 52 (62%)	
4	3 / 89 (3%)	0 / 37 (0%)	3 / 52 (6%)	
**BMI** (kg/m^2^)	22.68 (18.61, 26.19)	24.44 (19.65, 28.91)	20.96 (18.33, 25.21)	0.056
**Smoking**	45 / 89 (51%)	19 / 37 (51%)	26 / 52 (50%)	0.900
**Alcoholism**	19 / 89 (21%)	9 / 37 (24%)	10 / 52 (19%)	0.563
**Diabetes mellitus**	22 / 89 (25%)	8 / 37 (22%)	14 / 52 (27%)	0.568
**Hypertension**	42 / 89 (47%)	17 / 37 (46%)	25 / 52 (48%)	0.843
**Number of metastasis sites**	2.00 (1.00, 3.00)	2.00 (1.00, 2.00)	2.00 (1.00, 3.00)	0.326
**Site**				0.865
Breast	15 / 89 (17%)	8 / 37 (22%)	7 / 52 (13%)	
Colon	9 / 89 (10%)	4 / 37 (11%)	5 / 52 (10%)	
Lung	23 / 89 (26%)	10 / 37 (27%)	13 / 52 (25%)	
Others	29 / 89 (33%)	11 / 37 (30%)	18 / 52 (35%)	
Ovary	8 / 89 (9%)	2 / 37 (5%)	6 / 52 (12%)	
Stomach	5 / 89 (6%)	2 / 37 (5%)	3 / 52 (6%)	
**Hormone therapy**	6 (7%)	5 / 37 (14%)	1 / 52 (2%)	0.078
**Dose reduction of CTx**	18 (20%)	0 (0%)	18 (35%)	**< 0.001**
**Admission to ICU**	18 (20%)	7 (19%)	11 (21%)	0.796
**Orotracheal intubation or mechanical ventilation**	16 (18%)	7 (19%)	9 (17%)	0.845
**Central venous catheter**	29 (33%)	12 (32%)	17 (33%)	0.979
**Blood transfusion**	13 (15%)	4 (11%)	9 (17%)	0.392
**Cardiopulmonary resuscitation**	15 (17%)	7 (19%)	8 (15%)	0.661
**Previous lines of chemotherapy**	19 (21%)	7 (19%)	12 (23%)	0.637
**Hemoglobin**	9.99 (1.96)	10.53 (2.00)	9.61 (1.86)	**0.031**
**Hematocrit**	30.00 (5.75)	31.43 (6.06)	28.98 (5.35)	0.052
**Leukocytes**	12,200.00 (9,400.00, 15,100.00)	9,800.00 (7,700.00, 14,000.00)	13,550.00 (10,350.00, 17,125.00)	**0.002**
**Neutrophils**	8,820.00 (6,870.00, 13,000.00)	7,420.00 (6,200.00, 10,800.00)	10,285.00 (8,100.00, 14,060.00)	**0.012**
**Platelets**	296,000.00 (210,000.00, 407,000.00)	282,000.00 (203,000.00, 377,000.00)	318,500.00 (224,250.00, 456,750.00)	0.125
**Aspartate aminotransferase**	28.00 (19.00, 73.00)	24.50 (19.00, 40.75)	31.00 (19.00, 103.10)	0.198
(Missing data)	26	9	17	
**Alanine aminotransferase**	28.00 (13.00, 58.45)	24.00 (13.50, 35.50)	31.00 (12.50, 69.00)	0.387
(Missing data)	26	9	17	
**Total bilirubin**	0.40 (0.30, 1.25)	0.35 (0.20, 0.62)	0.60 (0.30, 1.55)	0.074
(Missing data)	26	9	17	
**Direct bilirubin**	0.20 (0.20, 1.10)	0.20 (0.18, 0.32)	0.40 (0.20, 1.45)	**0.046**
(Missing data)	26	9	17	
**Ionized calcium**	5.30 (5.00, 5.57)	5.30 (5.10, 5.60)	5.20 (5.00, 5.50)	0.054
(Missing data)	3	0	3	
**Creatinine**	0.70 (0.50, 0.90)	0.60 (0.40, 0.90)	0.70 (0.50, 1.02)	0.173
**Urea**	40.00 (25.00, 63.00)	31.00 (22.00, 50.00)	43.00 (31.00, 76.75)	**0.003**
**Creatinine clearance**	92.08 (63.95, 133.88)	120.68 (78.62, 157.36)	78.19 (57.02, 118.87)	**0.011**
**Uric acid**	3.90 (2.40, 4.55)	4.00 (2.05, 4.40)	3.75 (2.93, 4.78)	0.738
(Missing data)	66	30	36	
**Albumin**	2.90 (2.30, 3.40)	3.40 (2.98, 3.50)	2.40 (2.20, 3.00)	**0.001**
(Missing data)	52	21	31	
**Lactate dehydrogenase**	378.50 (218.75, 550.75)	374.00 (213.00, 464.50)	391.00 (259.00, 694.00)	0.261
(Missing data)	69	26	43	
** C-reactive protein**	71.50 (39.75, 156.25)	54.00 (27.00, 126.00)	96.00 (50.75, 172.50)	**0.010**
(Missing data)	1	0	1	

Notes: ^1^n / N (%); mean (standard deviation); median (interquartile range)

^2^Pearson’s Chi-squared test; Welch’s two sample t-test; Fisher’s exact test; Wilcoxon rank sum test; Wilcoxon rank sum exact test. CTx = chemotherapy, BMI = body-mass index, ECOG = Eastern Cooperative Oncology Group Performance Status, ICU = intensive care unit

Considering the two study groups—death or survival within 30 days of systemic oncological therapy—a statistically significant association with the worse outcome was found with a higher ECOG score (30-day mortality by ECOG category was 38.6%, 76.2% and 100% with ECOG 2, 3 and 4, respectively; p < 0.001); chemotherapy dose reduction (all 18 patients died within 30 days; p < 0.001); lower values of serum albumin (p = 0.001), hemoglobin (0.031), and creatinine clearance (0.011); and higher values of leukocytes (0.002), neutrophils (0.012), direct bilirubin (0.046), urea (0.003), and C-reactive protein (0.01; see Table 2).

No statistically significant associations were found between the main outcome and characteristics such as comorbidities, smoking, alcoholism, BMI, clinical staging, presence of metastasis in the central nervous system or any other site, admission to the ICU, or laboratory variables. There was also no association between the outcome and the presence or absence of oncological emergencies.

For the multivariate analysis, data such as the ECOG score, age, and BMI were included (due to the biological plausibility and the statistical significance found). This was in addition to the inclusion of other clinical and laboratory data (due to the finding of statistical significance in the univariate analyses), such as reduction in the dose of chemotherapy, serum albumin, hemoglobin, leukocytes, neutrophils, direct bilirubin, urea, creatinine clearance, and C-reactive protein. In the final model, only the albumin level remained statistically associated with the outcome (HR = 0.35; CI: 0.14, 0.90; p = 0.034; see Table 3).

**Table 3:** Predictors of death within 30 days of chemotherapy after multivariate analysis

Characteristic	HR	95% CI	p-value
**Age**	0.99	0.97, 1.02	0.625
**ECOG**			
2	—	—	
3 or 4	1.60	0.87, 2.92	0.126
**Body-mass index**	0.98	0.93, 1.04	0.552
**Hemoglobin**	1.01	0.85, 1.19	0.900
**Creatinine Clearance**	1.00	0.99, 1.00	0.219
**Albumin**	0.35	0.14, 0.90	**0.034**
**Direct bilirubin**	1.01	0.88, 1.16	0.842
** C-reactive protein**	1.00	1.00, 1.00	0.573
**Ionized calcium**	0.68	0.40, 1.17	0.152
**Previous lines of chemotherapy**	1.52	0.82, 2.82	0.180

Notes: HR = hazard ratio, CI = confidence interval, ECOG = Eastern Cooperative Oncology Group Performance Status.

## Discussion

It is well known that indiscriminate chemotherapy administration close to the end of life may be strongly associated with a greater number of invasive procedures preceding death, including ICU admissions, insertion of probes, deep venous access, and use of vasoactive drugs, among other interventions considered as “invasive measures” [8–11]. Some authors believe that chemotherapy in the last weeks of life is a low-quality factor in the assessment of patient care [12]. Nevertheless, urgent palliative chemotherapy is a treatment option for advanced disease in hospitalized patients of cancer, provided that there is some expectation of a positive response with an increase in survival or improvement of symptoms [3–5, 13].

One biggest challenge in oncology practice is knowing which patients can benefit from oncological treatment when hospitalized in a severe clinical condition. The ECOG-PS score can help make this decision by assessing the functional status and allowing a certain prediction of treatment toxicity in patients with cancer. Moreover, other criteria previously reported in the literature as predictors of worse prognosis and unfavorable outcomes can be considered for patients with severe cancer. These criteria include age, tumor sensitivity to chemotherapy, previous comorbidities, hypercalcemia, and hyperbilirubinemia [7, 12, 14].

Our results showed that patients hospitalized with advanced solid neoplasia and poor clinical performance had a mean post-chemotherapy survival time of 74 days, which is longer than that reported in the literature [7, 14–17]. Nevertheless, the number remains low, highlighting the poor prognosis of these patients, regardless of whether they receive systemic cancer treatment or complementary invasive measures during hospitalization. Another important point is that most previous studies used overall survival rather than post-chemotherapy survival as the main outcome. We consider that the option for the latter form was more appropriate in the temporal evaluation of these individuals, as it was possible to more reliably estimate the influence of an “urgent” chemotherapy regimen immediately after its institution. Given that all included patients would invariably die due to their advanced disease, we chose death or the last outpatient visit as the temporal endpoint. Surprisingly, this time interval exhibited great variability among the patients, reflecting the intrinsic differences between them.

We evaluated post-chemotherapy survival or death within 30 days in relation to the clinical characteristics of the patients and observed a statistically significant association between their ECOG performance status and a reduction in the prescribed chemotherapy. ECOG, a validated clinical performance scale, was related to lower survival, as expected. This association is consistent with the literature, with previous studies reporting a clear relationship between poor clinical performance and poor prognosis [2, 7, 12]. Reduction in the prescribed chemotherapy dose was associated with a lower survival rate. This finding may be explained as a confounding factor if we consider that physicians administering treatment during hospitalization tend to be more cautious when prescribing chemotherapy to frail patients or those with poor overall clinical performance. Nonetheless, this association makes us also hypothesize that even patients with poor clinical performance might benefit from a full-dose cancer treatment when hospitalized. To confirm this assumption and evaluate prolonged benefit, new prospective and controlled studies are necessary, since our study was not designed to answer this question.

We also found an association between post-chemotherapy early death and systemic arterial hypertension. Although no study reports a direct association between hypertension and worse survival, we believe that this reflects the worse survival reported in patients with comorbidities in general [7, 12].

There was an association between the outcome of death within 30 days after chemotherapy and the following laboratory variables: lower hemoglobin values, elevated direct bilirubin values, increased leukocyte and neutrophil counts, altered renal function with lower creatinine clearance and higher urea values, lower albumin, and higher C-reactive protein levels. These findings are consistent with the previous data on worse clinical outcomes associated with poor nutritional status (e.g., albumin levels), anemia, renal and liver dysfunction, and increased inflammatory marker levels [2, 7, 12, 18–21]. Another biomarker that has been recently studied as a prognostic factor in solid cancers is the lymphocyte-to-monocyte ratio, with a higher ratio related to longer overall survival [22–26]; however, we did not analyze this in our work, given that there is no ideal cutoff established to safely use it objectively. Our results are also in line with another prognostic tool previously established in medical literature for patients with advanced cancer, the Palliative Prognostic Score, in a multicenter study. Many of its clinico-biological variables were also present in our work and some predicted survival in the univariate analysis (albumin and leucocyte count, as examples), although we did not have the necessary statistical power to create a score [27].

However, in the multivariate analysis, variables with a statistically significant association in the binary evaluation lost their significance, with a positive association found for only albumin. This may have occurred due to the confounding factors and interactions between variables and may indicate a low power of association, perhaps due to the sample size, reinforcing the need for a new prospective and larger studies to further test these relationships.

As for the number of previous lines of chemotherapy, to our surprise, more than three quarters of the patients had not received systemic treatment previously (we call them “chemotherapy-sensitive”). We believe that these patients can be considered as being neglected by the system, because although they are part of the public healthcare system, their access to tertiary hospitals and medical subspecialties is often difficult and time consuming. These data motivated us to conduct an exploratory analysis of the subgroups of patients included in the study regarding whether they had previously undergone treatment; the aim was to assess whether there was a difference between the results of the tests performed. We observed that only “chemotherapy-sensitive” patients had lower survival rates with treatment dose reduction (i.e., there was a relationship between a lower dose and the worse outcome in only the first chemotherapy treatment), and with higher ECOG scores. These data suggest that treatment-naïve patients have worse outcomes and clinical performance; however, once the choice is made to prescribe chemotherapy to these hospitalized patients, it may not be worth reducing the dose. In the “polytreated” patient subgroup, greater survival was observed in patients with higher serum albumin levels, which may indicate that nutritional status plays a more important role in the final outcome for this subgroup.

In our study, approximately one-third of the patients were evaluated by the palliative care team during hospitalization. We questioned whether this low percentage was because we were being very “aggressive” in the treatment, viewing the patient primarily as a candidate for oncological treatments. This result even served as a point of reflection for improving our service, since we already have data in the literature relating to early access to the palliative care team to improve the quality of life and even ensure longer survival of patients with cancer [2, 7, 8, 28].

A limitation of this study is its retrospective observational nature, which made it difficult to establish causal relationships, and the lack of a control group that did not receive chemotherapy during hospitalization. We also had some variables with missing values in our database; fortunately, these values were similarly distributed among subgroups and, given the high importance of some variables, such as albumin, we opted to include them in the analysis, considering mostly the benefit from their inclusion through imputation. Ultimately, this profile of patients with poor clinical performance is not routinely addressed in clinical studies; thus, given the low-quality and scarce data in medical literature, we believe our results are relevant.

## Conclusion

Few previous studies address the recommendation of cancer treatment in critically ill or severely ill patients with low clinical performance; most of the time, these patients are excluded from clinical trials.

In this study, although we identified some predictive factors for 30-day survival after chemotherapy in the bivariate analysis, only the albumin level remained statistically significant in the multivariate analysis. Therefore, we still do not know which clinical and laboratory factors are effective and reliable predictors of a better outcome after the “last stand” chemotherapy. The elucidation of factors that truly predict the greater objective benefit of “heroic” or “urgent” oncological treatment for hospitalized patients in severe condition remains to be completely addressed. For more evidence-based decision-making in clinical oncology and, consequently, greater benefit for the patient, it would be of great value to develop a score in the future to predict the benefit of chemotherapy for patients who are critically ill. This score could include relevant clinical and laboratory variables to ease the decision-making between chemotherapy and standard care, by helping to exclude the subjective factor of the individual clinical impression of each physician. This would make their prescription to hospitalized patients with cancer much more assertive. Therefore, new prospective studies are required in this context.

## Data Availability

The dataset supporting the findings of this study is available in the repository: https://docs.google.com/spreadsheets/d/1xMHIX6nA4zYuZrSeoImKItUoUY2onRZB/edit#gid=1295960138.

## List of abbreviations

BMI: body-mass index
CI: confidence Interval
ECOG: Eastern Cooperative Oncology Group Performance Status
HR: hazard ratio
ICU: intensive care unit
M: presence or absence of distant metastases
N: number of lymph nodes
T: tumor size
